# Health Tract, No. 66

**Published:** 1865

**Authors:** 


### HEALTH TRACT, No. 66.

### URINATION.

CAREFULLY conducted and reliable experiments show, that when the thermometer is at seventy, and the air is fine, dry, and clear, a healthy adult will pass something less than three pints of urine in twenty-four hours; but he will pass six pints if the day is raw and windy, the atmosphere saturated with dampness, and is several degrees cooler.

On the other hand, it is found, that on a beautiful, clear day, six pints of fluid are passed from the skin and lungs, and but four pints on a damp, raw day. That is, on fair days, thirty-eight per cent of the fluids passed from the system is in the shape of urine; and sixty-two per cent by skin and lungs. On damp, raw days, seventy-one per cent is in urine, and twenty-nine per cent in perspiration. Every observant person knows that he does not feel as lively, cheerful, and buoyant in raw, damp weather, as when it is clear and dry. The reason of this is, that counting a pint a pound, there is in a damp day, one or more pounds of matter in the system than there ought to be: it is then no wonder that on such days we feel heavy, depressed, dispirited, and gloomy. In fine weather, this matter, for which the system has no further use, passes steadily from the body as fast as it accumulates, and we feel elastic in body and in mind, buoyant, and cheerful. In damp, raw, windy, and cooler weather, the pores of the skin are closed by these four agencies; the waste fluids can not pass in this direction, but must find exit, in greater part, through the bladder, to be emptied at varying intervals. It follows, then:

First. The warmer the weather, the greater the perspiration, and the less the urine.

Second. As exercise promotes perspiration, the more exercise, the less urination.

Third. Hence, unequal amounts of urination from day to day, do not necessarily indicate disease; for it is Nature regulating the "waste ways" of the system.

Fourth. As persons feel best when the pores of the skin are open, free and soft, as in perspiration, the surface of the body should be kept soft, warm, and clean, as a means of health and that general feeling of wellness which happifies the heart.

Fifth If, in dull, damp weather, the system is burdened by a pound or more of fluid substances which ought to be out of it, almost the entire amount of discomfort engendered by it could be readily avoided, by eating and drinking one half less on such days than on others; that is, about a pound and a half, instead of three pounds, in twenty-four hours.

Sixth. As we naturally perspire less in damp, raw, cold, windy weather, it is the dictate of wisdom to excite perspiration artificially by steady labor, or active exercise in the open air.

But the great misfortune is, that instead of eating less, and exercising more in bad weather than usual, we exercise less, because we are afraid of the weather, and we eat more because we have nothing else to do, and being the only source of pleasure, we yield ourselves more completely to it. The same reasoning is applicable to the Sabbath-day—to wit, exercising but little, we should eat but little

